# Impact of Pneumococcal Conjugate Vaccines on the Incidence of Pneumonia in Hospitalized Children after Five Years of Its Introduction in Uruguay

**DOI:** 10.1371/journal.pone.0098567

**Published:** 2014-06-06

**Authors:** María Hortal, Miguel Estevan, Miguel Meny, Inés Iraola, Hilda Laurani

**Affiliations:** 1 Basic Sciences Development, National University, Montevideo, Uruguay; 2 Radiology Department, Children's Hospital, Ministry of Health, Montevideo, Uruguay; 3 Statistics Department, Montevideo Municipality, Uruguay; 4 Pediatric Department, Social Security, Montevideo, Uruguay; 5 Immunization Department, CHLAP, Montevideo, Uruguay; Centers for Disease Control & Prevention, United States of America

## Abstract

**Background:**

Data on the burden of pneumococcal disease and the most frequent serotypes demonstrated that invasive disease and pneumonia were important manifestations affecting children under 5 years of age. Therefore, pneumococcal diseases prevention became a public health priority. Uruguay was the first Latin American country to incorporate PCV7 into its National Immunization Program. The aim of this study is to compare the incidence rates for hospitalized pneumonia in children from the pre PCV introduction period and the following five years of PCVs application in Uruguay.

**Methods and Findings:**

Population-based surveillance of pneumonia hospitalization rates, in children, less than 14 years of age, had been performed prior pneumococcal vaccination, and continued following PCV7 introduction and PCV13 replacement, using the same methodology. Hospitalized children with pneumonia were enrolled from January 1, 2009 through December 31^st^, 2012. The study was carried out in an area with a population of 238,002 inhabitants of whom 18, 055 were under five years of age. Patients with acute lower respiratory infections for whom a chest radiograph was performed on admission were eligible. Digitalized radiographs were interpreted by a reference radiologist, using WHO criteria. Pneumonia was confirmed in 2,697 patients, 1,267 with consolidated and 1,430 with non consolidated pneumonia of which incidence decrease, between 2009 and 2012, was 27.3% and 46.4% respectively. 2001–2004 and 2009–2012 comparison showed a significant difference of 20.4% for consolidated pneumonia hospitalizations. A significant incidence decline was recorded among children 6 to 35 months of age.

**Conclusions:**

An overall significant reduction in pneumonia hospitalizations was observed following the introduction of PCV7 and furthermore following the change to PCV13.

## Introduction

*Streptococcu pneumoniae* is an important cause of severe morbidity and mortality among the pediatric population. Following efficacy studies, the heptavalent pneumococcal conjugate vaccine (PCV7) was approved for use in the pediatric population in 2000. It was introduced into National Immunization Programs (NIPs) globally, in order to control pneumococcal diseases in the most vulnerable age groups, particularly those less than 24 months of age. PCV7 effectiveness was demonstrated for the prevention of vaccine type pneumococcal diseases, as well as, for all cause pneumonia and otitis media.Despite its effectiveness, expanding serotype coverage led to the introduction of PCVs with more serotypes. PCV13, which contains the 7 original serotypes and six additional serotypes all conjugated to the carrier protein, CRM197, approved in 2010.

Data on the burden of pneumococcal disease, and the most frequent serotypes involved, demonstrated that invasive disease, as well as, pneumonia were the most important manifestations affecting Latin American children less that 5 years of age [1]–[3].

Uruguay was the first Latin American country to incorporate PCV7 into its NIP in a 2+1 dosing schedule. PCV7 was incorporated in March 2008, with doses given at 2, 4 and 12 months of age, for the 2008 birth cohort, and a catch-up program of 2 doses, at 15 and 17 months of age, was given to the 2007 birth cohort. In March 2010, PCV13 replaced PCV7, with the same dosing schedule, and a catch-up was offered to children up to 5 years of age.

Population-based surveillance of pneumonia hospitalization rates in children less than a month to 14 years of age, had been performed prior to initiation of PCV7 vaccination, and continued following the introduction of PCV7 and PCV13, using the same methodology [4], [5]. The aim of this study is to compare the incidence rates for hospitalized pneumonia in children from the pre PCV introduction period, and the following five years of its application in Uruguay.

## Methods

This population-based surveillance, enrolled hospitalized children with community-acquired pneumonia, from January 1, 2009 through December 31^st^, 2012. The study was carried out in two municipalities of the northwest region of Uruguay, that according to the 2011 National Population Census a population of 238,002 inhabitants was registered, of whom 60 464 (25.4%) represented the pediatric population (0 to 14 years of age). Of those 18,055 (7.6%) were less than five years of age.

Although the pneumonia burden predominated among children under 5 years of age, our surveillance pre and post pneumococcal vaccination, enrolled patients with all pediatric ages [5].

Previously published data were used to compare incidence rate pre- PCV introduction and post-both PCV7 and PCV13, based on the binomial distribution (asymptotic). Same methodology was employed for 2001–2004 and 2009–2012 surveillances. The four year interruption of the surveillance was due to the delayed decision for PCV implementation. March 2008, PCV vaccination was launched for the birth cohort of the same year and, a two doses catch-up, was offered to the 2007 cohort at 15 and 17 months of age. In January 2009, surveillance of pneumonia in hospitalized children started. Patients aged 0 to 14 years with community-acquired acute lower respiratory tract infections, for whom a chest x-ray was performed on admission, and were hospitalized at one of the four hospitals (2 public and 2 private) available within the study area, were eligible. Pneumonia surveillance on hospitalized children began January 1^st^, 2009 and ended December 31^st^, 2012 [6]. Hospital-acquired pneumonias were excluded, as were other respiratory conditions (bronchiolitis, asthma/bronchial hypersensitivity, etc.), in which no chest radiograph was ordered. Data were abstracted from patient medical records. Patient's vaccination certificate provided information on PCV7/PCV13 doses and date of vaccine administration.

Chest x-rays were digitalized and interpreted by a reference radiologist, blinded to the clinical diagnosis. Radiographs were interpreted using WHO criteria, which consists of alveolar consolidation or pleural effusion and non-consolidated pneumonia with mild interstitial/perihiliar changes[6]. PCVs were universally recommended and provided free of charge. Since March 2008, PCV7 was administered, but in April 2010, was replaced by PCV13, either used to complete schedules initiated with PCV7 or to vaccinate by cohorts, starting with the 2010 birth cohort. Both vaccines used a 2+1 dosing schedule: given at 2 and 4 months of age and a booster when 12 months old. Additionally a single dose catch-up program was carried out for birth cohorts 2005 through 2008, covering children <5 years of age. Children who had received one or two PCV7 as part of primary series prior to PCV13 introduction completed their vaccination schedule with PCV13. According to NIP information vaccination compliance, with at least one vaccine dose, by the end of the observation period, was 97.7% for PCV7 and 99.8% for PCV13.

Bacterial etiology in blood and pleural aspirates was investigated as per routine medical practice. *S. pneumoniae* isolates were referred to the National Reference Laboratory for identification confirmation, and serotyping was performed using the Quellung reaction. Viral etiology for influenza A/B, RSV and adenovirus was investigated in nasopharyngeal aspirates (NPA) by rapid techniques (immunochromatography or/and direct immunofluorescence).

Compliance with study protocol was assessed by periodic audits. The study data were sent to the study headquarters group in Montevideo, at the Comisión Honoraria de Lucha Antituberculosa y Enfermedades Prevalentes (CHLAP), for quality control. A database was collected into Epi-Info, CDC 2002 and statistical analysis was performed using SPSS statistic package. The study protocol was approved by the National Ethics Committee of the Ministry of Health, and endorsed by the Minister of Health who signed the communicate.

## Results

### Study population

During the surveillance period (2009–2012), 3,677 patients were enrolled. As per enrollment criteria, all patients had chest radiographs performed and pneumonia was confirmed in 2,697 (73.3%) cases. Patients less than five years of age, represented 77.4% of the eligible patients. The enrollment averaged 919 cases per year and, the average confirmed pneumonia cases, was 674 per year, although yearly variations occurred. Table 1 provides the number of pneumonias (consolidated and non-consolidated) enrolled per years pre and post PCV implementation and their estimated incidences for hospitalized children due to pneumonia. Comparison 2009 to 2012 incidences of consolidated and non-consolidated pneumonias revealed a reduction of 27.3% and 46.4% respectively, meanwhile comparison of 2001–2004 with 2009–2012 showed significant difference of 20.4% for consolidated pneumonias and non-significant for non-consolidated pneumonia (2.2%).

**Table 1:** Total enrolled cases, incidence rates, x-ray classification by years pre and post PCVs implementation.

	Consolidated Pneumonia		Non Consolidated Pneumonia	
Years	Cases	Incidence*	Cases	Incidence*
2001–04	1062	585	1081	596
2009	428	708	481	796
2010	292	483	358	592
2011	236	390	333	551
2012	311	514	258	427

*Incidence is calculated by 100,000 person-year.

### Impact of PCVs on pediatric hospitalizations due to pneumonia

The incidence rate of consolidated pneumonia post vaccination showed significant decreases among patients aged 12 to 35 months, if compared with the incidence rate for the pre-vaccination in the same age group (Table 2). The non-significant comparison result for infants 0 to 11 months of age was unexpected, however disaggregating the data into 0 to 5 months and 6 to 11 months, a significant incidence reduction (2737/1982/10^ 5^ person-year), was assessed for the older group. Consequently, PCV7 and PV13 impact was observed among children aged 6 to 35 months. Incidence reduction of consolidated pneumonia in children 6 to 11 months of age was 27.6%, while among those aged 12 to 23 months and 24 to 35 months, decline was 37.8% and 23.7% respectively.

**Table 2:** Consolidated pneumonia hospitalizations: cases and incidence pre and post by age groups.

Pre vaccination				Post vaccination		
Age (months)	Population at risk	n	Incidence	n	incidente	P Value
0–11	3507	274	2604	333	2374	NS
Dic-23	3497	250	2383	207	1482	<0,001
24–35	3584	145	1349	147	1029	<0,05
36–47	3658	89	811	97	663	NS
48–59	3809	77	674	101	663	NS
Total	18055	835	1542	886	1227	<0,001

Table 3 compares incidence among pediatric population targeted for vaccination, with incidence of older children not targeted for vaccination.

**Table 3:** Incidence comparison between vaccine targeted and non-targeted age groups.

		Pre-vaccination		Post-vaccination		
Age (months)	Population at risk	n	incidence	n	incidence	p value
0–35	10588	669	2106	688	1624	<0,001
36–59	7467	166	741	198	633	NS
Total	18055	835	1542	886	1227	<0,001

**Figure 1:**
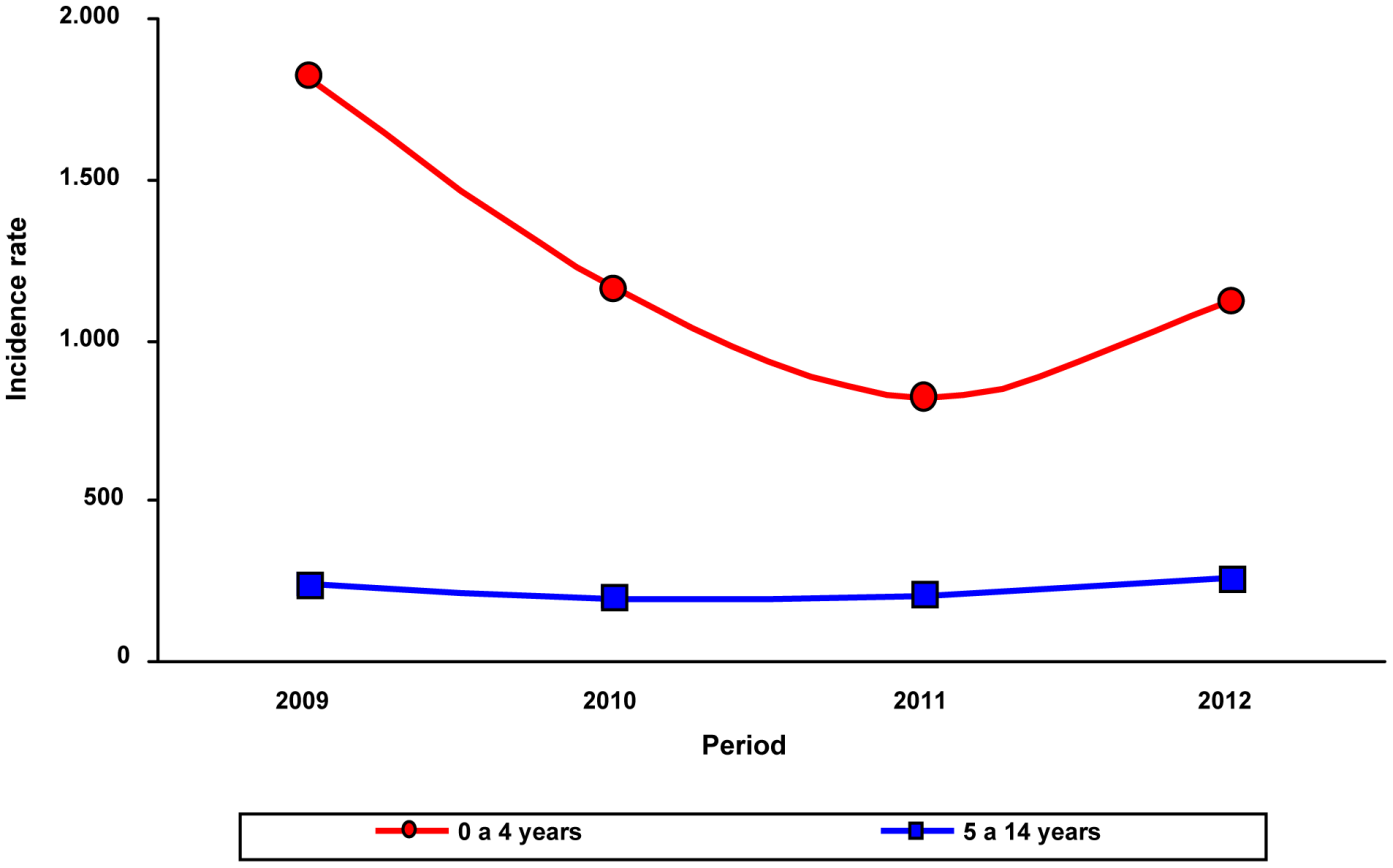
Consolidated pneumonia incidence: comparison between children aged 0–4 years versus those 5–14 years, by observation years.

**Figure 2:**
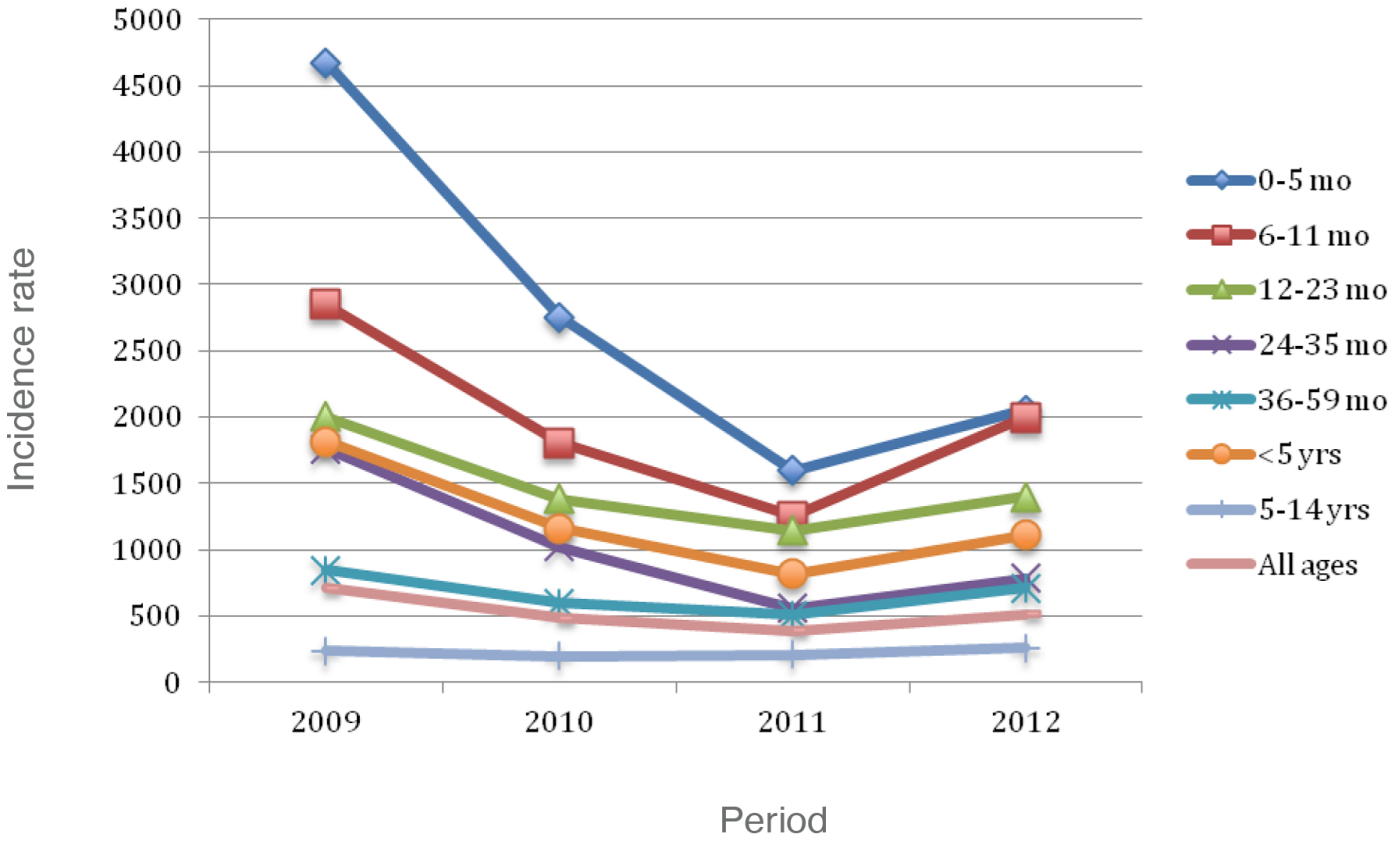
Consolidated pneumonia incidence per 10^5^ by age groups and observation years.

**Figure 3:**
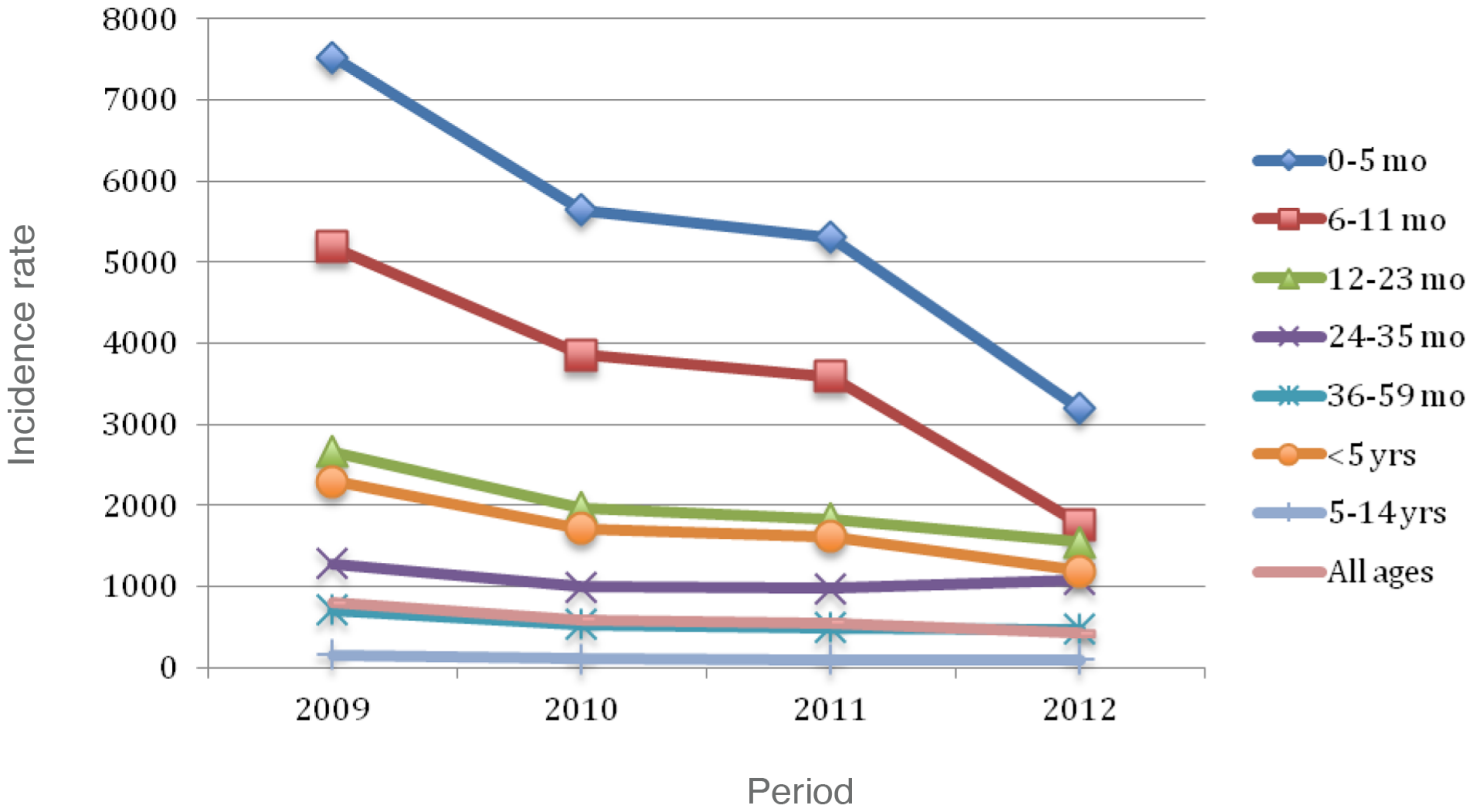
Non-consolidated pneumonia incidence per 10^5^ by age groups and observation period.

Figure 1 shows, by years of surveillance (2009–2012), the.incidence of patients 0 to 4 years, versus the incidence of those aged 5 to 14 years. Figures 2 and 3 present consolidated pneumonia and non-consolidated pneumonia incidences respectively, by age groups and years of post-PCVs application.

Reductions were seen yearly for all children <5 years of age, as well as, for all age subgroups, with exception of 2012, where an increase was seen. Despite this transient increase, an overall significant decrease was seen when comparing incidences from 2009 to 2012. No consistent or statistically significant change in consolidated pneumonia occurred in children 5–14 years of age. Otherwise, a significant decrease (48.2%) for non-consolidated pneumonia, was recorded among patients aged <5 years.

### *S pneumoniae* and viral etiologies

During the post PCV, *S pneumoniae* etiology was confirmed in 81 of the 2,697 patients, 78 (3.0%) had consolidated pneumonia, of which 34 demonstrated a pleural effusion, and 3 had non-consolidated pneumonia. *S. pneumoniae* was isolated in 70 specimens and latex was positive in 11. Forty-three of the 70 (61.4%) isolates were available for serotyping; the majority cultured from clinical samples of children <5 years of age. Table 4 shows the serotypes divided in two age groups (<5years and older) by years of surveillance. Serotype 1 was the most frequent (15/43) in both age groups. Eleven non-vaccine serotypes were found, almost exclusively among the youngest.

**Table 4:** *S pneumoniae* serotypes by age groups and years.

Year	<5 years	5–14 years
2009	1, 3, 5(2), 6A, 7F, 14, 19A(2), 23F	5
2010	1(3), 7F, 12F(2), 14, 23F, 33F	1(3), 3
2011	1(3), 3, 10A, 14, 23F, pool I (2)	1(3), 3
2012	1, 9N, 12F, 24A	1, pool C

Viral etiology was sought in NPA of 1,734 patients, 499 with consolidated pneumonia, 789 with non-consolidated pneumonia, and 446 without pneumonia. Virology was positive in 166 patients with consolidated pneumonia (32.1%) and in 284 with non-consolidated pneumonia (36.0%).Viruses identified were as follows: influenza A (34), RSV (113) and adenovirus (13) in consolidated pneumonia patients and influenza A (34), RSV(239) and adenovirus (11) in non-consolidated pneumonia patients. A coinfection with adenovirus/*S pneumoniae* was found in one patient with non-consolidated pneumonia. In 2012, whooping cough syndromes were diagnosed in 20 patients under 12 months of age. Their chest radiographs revealed non-consolidated pneumonia in 8 patients, one consolidated pneumonia and 11 no pneumonia..

## Discussion

Although pneumonia is a major cause of morbidity and mortality globally, precise microbiologic diagnosis is difficult. To assess the burden of pneumonia on pediatric population the WHO developed guidelines for standardized interpretation of chest radiographs [6]. Bacterial etiology was associated with consolidated pneumonia, (sometimes complicated with pleural effusion), which by frequency was presumed to be pneumococcal, in countries where conjugate *H influenzae* type b vaccination was in use since the nineties [7]. Therefore, pneumococcal pneumonia is one of the most important bacterial cause of community-acquired pediatric pneumonia leading to hospitalization and its control is a public health priority [8]. Surveillance of hospitalizations for pneumonias, in addition to specific rates of microbiologically confirmed pneumococcal pneumonia, are appropriate endpoints to evaluate the impact of PCV programs. Comparison of hospitalization rates for community-acquired pneumonia and pneumococcal pneumonia between pre-vaccine years (2005–2007) and the year after vaccination (2009) decreased significantly in all children by 56% and 42.8% respectively, at the Children's Hospital in Montevideo [9].

Population-based pneumonia incidence is available from a selected area in Uruguay. A three years pre-vaccination study, demonstrated an average annual consolidated pneumonia incidence in children aged 12 to 23 months of 1,175/10^5^ person-year [ Same surveillance protocol was applied to evaluate the impact of PCV7 and used in this study to evaluate the continued impact of PCV7 and the impact following switch to PCV13.

Four years of surveillance of the five years post introduction of PCVs, demonstrates significant reductions in consolidated pneumonias that by the end of 2012 involved the main vaccine targeted age groups (6–35 months). Incidence reduction of consolidated pneumonias in children 12 to 23 months of age predominated (37.8%), although children aged 24 to 35 months showed a significant decline not observed in the previous study [8]. However, despite high coverage, when vaccinating by birth cohorts the decrease progress is slow, different from what happens in other countries. In U.S., after six years of PCV7 introduction, 35% decline was observed for all-cause pneumonia hospitalizations in children younger than 34 months of age [10]. Recently a population-based surveillance for hospitalized children due to alveolar pneumonia, in Israel, revealed 40.6% of PCVs effectiveness [11]. Furthermore, among hospitalized Canadian children, 72% decline for lobar pneumonia admissions was detected [12].

A 2009–2012 decline, was also reported for non-consolidated pneumonias in children in the vaccine age group (<2 years of age), traditionally thought to be of viral etiology [13]–[16]. Viral cases, apparently controlled by PCVs, have already been described by several authors in both efficacy and effectiveness studies of CRM_197_ conjugated vaccines [13]-[17]. This observation may point to the general fluctuation of yearly viral infections in all age groups. Of interest is the absence of change in hospitalizations for consolidated pneumonia in the 5–14 year age group, thus in this study an indirect effect of PCVs on pneumonia hospitalizations, was not evident.

Confirmation of pneumococcal etiology was very infrequent, with only 70 isolates and 43 strains available for serotyping. Overall relatively few non-vaccine serotypes were identified, and the vaccination status shows decreasing vaccine failures while coverage progresses.

We saw a yearly decrease in hospitalizations for pneumonias in our study population, with exception of 2012, in which increases were seen, but despite these, the decreases were significant. The cause of this increase is unclear, and since it was not seen in non-consolidated pneumonias it is unlikely to be due to an increase in circulating viral agents. We investigated potential changes in evaluation or hospitalization of patients with suspected pneumonia, and found no change which would explain the increase in 2012. Other possible explanation, may be, an increase in other bacterial etiologies such as *S aureus* or non-vaccine pneumococcal serotypes. Of interest is the increase in whooping cough symptoms in young patients in 2012, some of whom had radiographic consistent with pneumonia. *B pertussis* which is occasionally cause of bacterial superinfections, particularly in young infants, but as the increase was seen in 2012 patients of all ages it would be unlikely to be the sole explanation. Despite the slight increase in 2012, there was overall significant reductions in pneumonia hospitalizations following the introduction of PCV7 and furthermore following the change to PCV13. Although, this ecological study does not prove cause and effect, it mirrors data obtained post PCV7 and PCV13 observed in other countries, using both the 3+1 and 2+1 dosing schedules, suggesting the role that these vaccines have in reduction of pneumonia hospitalizations [18], [19]. Ongoing surveillance is required to continue to monitor changes in pneumonia, as well as, other outcomes following PCV vaccination.
